# The GREENH-City interventional research protocol on health in all policies

**DOI:** 10.1186/s12889-017-4812-8

**Published:** 2017-10-18

**Authors:** Marion Porcherie, Zoé Vaillant, Emmannuelle Faure, Stéphane Rican, Jean Simos, Nicola Luca Cantoreggi, Zoé Heritage, Anne Roue Le Gall, Linda Cambon, Thierno Amadou Diallo, Eva Vidales, Jeanine Pommier

**Affiliations:** 1 EHESP –School of Public Health, Department of Social Sciences and Health, 15 avenue du Professeur Léon-Bernard - CS74312 -, 35043 Rennes cedex, France; 2 ARENES, (UMR/CNRS 6051), University of Rennes 1 Institut d’Etudes Politiques, 104 Boulevard de la Duchesse Anne, 35700 Rennes, France; 3 University of Paris-Nanterre, Ladyss - UMR 7533, 200 Avenue de la République, 92000 Nanterre, France; 4 Institute of Global Health, University of Geneva, Chemin des Mines 9, CH - 1202 Genève, Switzerland; 5 WHO French Healthy City Network, 15 avenue du Professeur Léon-Bernard - CS74312, 35043 Rennes, France; 6 EHESP –School of Public Health, Department of environmental and occupational health and sanitary engineering, 15 avenue du Professeur Léon-Bernard - CS74312, 35043 Rennes cedex, France; 7 EHESP –School of Public Health, INCA/EHESP Research Chaire in Cancer Prevention, Department of Social Sciences and Health, 15 avenue du Professeur Léon-Bernard - CS74312 -, 35043 Rennes cedex, France; 8 École supérieure d’aménagement du territoire et de développement régional– Université Laval, Pavillon Félix-Antoine-Savard, bureau FAS-1616, 2325, allée des Bibliothèques, Québec, QC G1V 0A6 Canada; 9 EHESP – National School of Public Health, Department of Social Sciences and Health, 15 avenue du Professeur Léon-Bernard - CS74312, 35043 Rennes cedex, France

**Keywords:** Health in all policies, Urban green spaces, Health inequities, Mixed-methods, Comparative multiple-case studies, Interventional research, Transferability

## Abstract

**Background:**

This paper presents the research protocol of the GoveRnance for Equity, EnviroNment and Health in the City (GREENH-City) project funded by the National Institute for Cancer (Subvention N°2017–003-INCA). In France, health inequities have tended to increase since the late 1980s. Numerous studies show the influence of social, economic, geographic and political determinants on health inequities across the life course. Exposure to environmental factors is uneven across the population and may impact on health and health inequities. In cities, green spaces contribute to creating healthy settings which may help tackle health inequities. Health in All Policies (HiAP) represents one of the key strategies for addressing social and environmental determinants of health inequities. The objective of this research is to identify the most promising interventions to operationalize the HiAP approaches at the city level to tackle health inequities through urban green spaces. It is a participatory interventional research to analyze public policy in real life setting (WHO Healthy Cities).

**Method/design:**

It is a mixed method systemic study with a quantitative approach for the 80 cities and a comparative qualitative multiple case-studies of 6 cities. The research combines 3 different lens: 1/a political analysis of how municipalities apply HiAP to reduce social inequities of health through green space policies and interventions 2/a geographical and topological characterization of green spaces and 3/ on-site observations of the use of green spaces by the inhabitants.

**Results:**

City profiles will be identified regarding their HiAP approaches and the extent to which these cities address social inequities in health as part of their green space policy action. The analysis of the transferability of the results will inform policy recommendations in the rest of the Health City Network and widely for the French municipalities.

**Discussion/conclusion:**

The study will help identify factors enabling the implementation of the HiAP approach at a municipal level, promoting the development of green spaces policies in urban areas in order to tackle the social inequities in health.

**Electronic supplementary material:**

The online version of this article (10.1186/s12889-017-4812-8) contains supplementary material, which is available to authorized users.

## Background

**Fig. 1:**
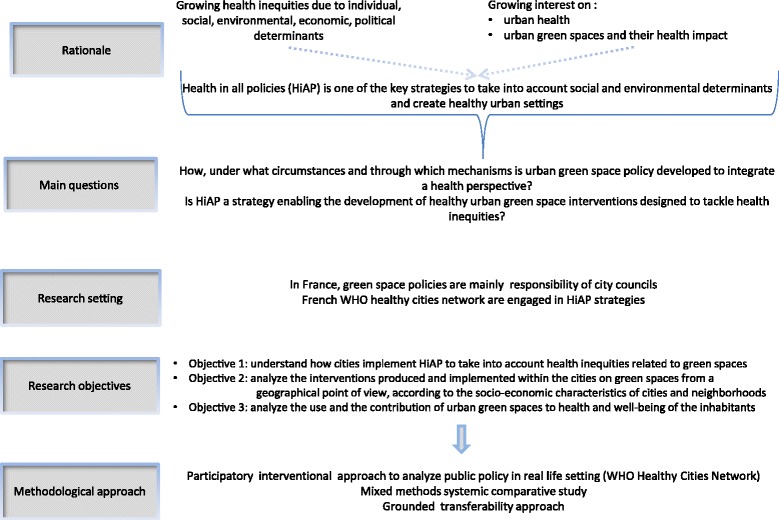
Rationale, research setting, objectives and methodological approach of the GREENH-city project

This paper relates the protocol of the GoveRnance for Equity, EnviroNment and Health in the City (GREENH-City) project funded by the French National Institute for Cancer (INCa – N°RI-2017-003). This research aims to identify the most promising interventions to operationalize the approaches of Health in All Policies at the city level to tackle health inequities through urban green spaces. Figure 1 presents the rationale and the objectives of the project.

Health inequities are much larger in France than in most other European countries even though its health system was considered in the early 2000s as one of the best in the world. Today it ranks 15th on a world scale [1]. Health inequities arise from a multitude of health determinants which go beyond health systems themselves. As noticed by the Marmot commission, these inequities are “seen in the conditions of early childhood and schooling, the nature of employment and working conditions, the physical form of the built environment, and the quality of the natural environment in which people reside. Depending on the nature of these environments, different groups will have different experiences of material conditions, psychosocial support, and behavioural options, which make them more or less vulnerable to poor health” [2] .

Numerous studies exist today showing the influence of social, economic, geographic or political factors across the life course as determinants of health [3–7]. These determinants affect individuals unevenly and thus create health inequities [8].

### The effect of environmental factors

Early research into health determinants often dealt with health behaviors (eating habits, alcohol consumption, smoking, sports etc.) and their link with non-communicable diseases including cancer. More recently, research has started looking into other health determinants and the role of environmental factors such as exposure to air pollution and heavy metals.

Environmental determinants are not necessarily risk factors; they may also offer health benefits [9]. For example some studies have shown that green urban spaces support health living for city dwellers [10–14].

### Green spaces in the built environment

As urban environments expand, new health challenges arise for the population living in them [15]. In this respect urban green spaces can help improve living conditions and influence people’s health and wellbeing [16–20]. The proximity and access to green space offers both physical and mental health benefits. A recent WHO review has shown for example that immune systems may be enhanced by the relaxation provided in green spaces [14]. Green spaces act on cognitive functions and mental health. They also have an impact on chronic diseases such as type 2 diabetes, hypertension, cardiovascular disease and certain cancers [10, 12, 21].

Furthermore, urban green spaces encourage healthier behaviors such as physical activities. They also provide recreational settings and promote social cohesion [22]. Lastly, their features, type and size all contribute to regulating urban ecosystems by depolluting the air, and reducing noise levels and the heat island effect [23].

Some studies have shown that health benefits provided by green spaces affect individuals to varying degrees according to age, gender, physical condition and social position [24, 25]. For example, these effects are thought to be greater on people belonging to lower social categories. The reason for this may relate to higher levels of physical, mental and social vulnerability than in upper social categories, or differences in exposure to the characteristics of green spaces: physical features which are either salutogenic (protective exposure) or pathogenic (exposure to harmful substances or air pollution) [13].

### Green space management policies

Some green space management policies can be detrimental to health. This includes the use of phytotoxic products, pesticides and herbicides which also has an adverse effect on biodiversity [11, 17].

However other policies have adopted a more sustainable approach to managing their green spaces. Such approaches can offer positive health outcomes through the mechanisms described above [11, 16, 24, 26, 27].

To understand how urban green spaces affect people’s health, we need to study their accessibility, management and features at the same time. There is, however, great variability in public policy across cities and districts, which leads to different levels of green space access and thus to health inequities [28, 29]. There are many definitions of urban green space in the literature which may or may not include: public parks, closed public green spaces (school playgrounds etc.), private gardens, blue spaces, play areas, allotments [14]. All may have some effect on people’s health.

### Urban green spaces and health: Where health in all policies (HiAP) come into play

New forms of policy engagement need to be introduced if health issues are to be addressed by sectors that traditionally are not directly concerned with health, such as environmental planning and green spaces.

In this respect, the HiAP approach offers a promising framework [30–33] for integrating health into other areas of policy as a way to support the wellbeing of the population and to achieve equity in health [34–37]. HiAP is important for acting on the social and environmental determinants that affect people’s living conditions and health inequities [38]. The most appropriate level at which to implement this approach would seem to be the local level [39]. In France, urban green space policies and management are principally the responsibility of towns and cities councils. Although there is an increasing amount of international research into urban green spaces and their effects on health, little research has been conducted on the policy choices made by cities with regard to green space management and their potential impact on health inequities.

The research protocol of the GREENH-City project therefore explores a relatively new area. The setting of the research is based in the French WHO Healthy Cities Network, whose members are committed to implementing HiAP to address health inequities and urbanism.

### Research questions and objectives

Considering this background, the project seeks to answer the following questions: what policies are French cities developing with regard to green spaces and health? How do these policies address health inequities and HiAP? What are the contexts and the right conditions for rolling out these policies at a local level? How does the population use or not use these urban green spaces? Does the whole population have access to these urban green spaces and in what conditions? And lastly, to what extent do these contexts and policies enable health inequities to be tackled?

Using mixed methods, this study explores the policy making processes which are favorable to health and the links with green space policy and health inequities. Bauman’s framework (2014) serves as a general framework to identify the HiAP process and the cities’ involvement in green space policy [40]. Within this framework, different levels of the HiAP approach can be analyzed: policy design, policies developed and synergies identified, relevant policies implemented, indicators of success, population outcomes. As such, the research objectives are to:

- understand how the cities implement HiAP in order to take into account the health inequities thanks to the political decisions related to green spaces (Objective 1)
- describe and analyze the interventions produced and implemented within the cities on the green spaces from a geographical point of view, according to the socio-economic characteristics of cities and neighborhoods (Objective 2)
- analyze the use and the contribution of urban green spaces to health and well-being of the inhabitants (Objective 3).

This study explores how public policy is actually implemented in the cities of the French WHO Healthy Cities Network. It is a real life interventional study in which various situations will be compared and contrasted to identify the best configurations for addressing health inequities in developing or maintaining urban green spaces.

The PRISMA-P 2015 checklist items relevant for this type of study are used to present this protocol.

## Methods/design

The project will be implemented by a multidisciplinary team: political sciences, social geography, urban planning and public health. Different institutions and stakeholders are involved in the project: researchers from two universities EHESP-School of Public Health and University of Paris Nanterre, practitioners from the WHO French Healthy Cities Network and experts with experience in HiAP (Institute of Global Health of Geneva University and École supérieure d’aménagement du territoire et de développement régional de l’Université Laval).

### Setting of the research

In France, cities are legally responsible for organization of public services such as crèches and retired people’s homes, transport, spatial planning and local development, which may strongly impact the urban environment. Health policies come mainly under the jurisdiction of other institutions (National and regional health authority authorities). Some cities choose, for historical reasons, or by political will, to actively promote health issues in addition to their legal mandates.

### Study design

**Fig. 2:**
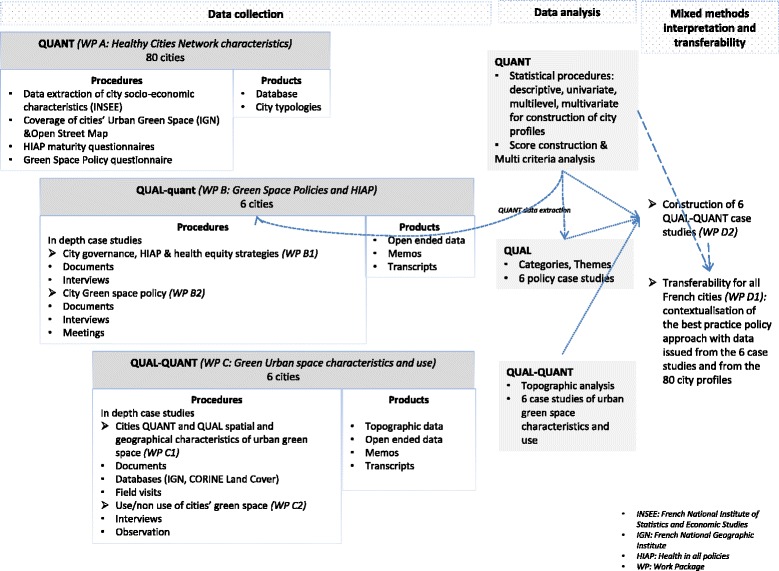
Mixed methods methodological approach: data collection, analysis and interpretation procedures and products *(QUAN = quantitative; QUAL = qualitative)*

This mixed-method research combines a large quantitative study alongside in-depth qualitative case studies [41] (See Fig. 2).

The case study design is used because we seek to understand the contextual conditions which we think will influence the phenomena studied. By combining multiple data sets, it should provide complete and in-depth picture of the phenomena [42].

#### The sample

This research will based on city members of the French WHO Healthy Cities Network, set up on the initiative of the WHO Regional Office for Europe. The French Healthy Cities Network, which is one of the most developed and structured networks in Europe, has been chosen as a sample for this study as it covers health issues across various fields, policies and programs conducted by the city members. The members of the French Healthy Cities network take a keen interest in health and health inequities. As such, they can provide a setting for research to identify policy action in health that is conducive to reducing health inequities. The Network has to develop HiAP at a local level. These principles were recently reaffirmed by the Declaration of [43] which strengthens the leadership of cities in promoting health and wellbeing and in tackling inequities. Adhering to the Athens Declaration represents a strong political statement and all network members agree to uphold it. Therefore, this network has been identified as being most suitable for the purposes of our study since member cities and conurbations do, theoretically, implement HiAP already.

First of all, a large quantitative study will be conducted across the 80 city members. These cities are considerably varied in terms of their populations, their political orientation (right wing/left wing) and urban configuration (from 6000 to over 2 million inhabitants). Among the 80 members, a sub-sample of 6 cities will be identified for an in-depth comparative study.

#### Research proceedings

The project has been divided into work packages which can be undertaken concomitantly:

- Work package A involves a baseline study to analyze the characteristics of the Healthy Cities Network.
- Work package B investigates objective 1: Understand how cities implement the approach of health in all the policies, in order to take into account the health inequities thanks to the political decisions related to green spaces,
- Work package C investigates objectives 2 and 3: Describe and analyze cities’ interventions produced and implemented on the green spaces from a geographical point of view, according to the socio-economic characteristics of the cities and neighborhoods and analyze the use and the contribution of urban green spaces to health and well-being of the inhabitants.
- Work package D involves cross-analysis of data and proposed recommendations.

More details about the work packages are provided below including their aims, the dimensions studied and the theoretical frameworks underlying the methods used. A table of the workpackages explaining for each one of them the objectives, method, population, analyzed dimensions, tools and deliverables is presented in Annex 1 (See Additional file 1 presenting Objectives and Methods by Workpage).

- Work package A (QUANT)**:** Baseline study: Healthy Cities network characteristics (*N* = 80 cities)

A large quantitative study (see Table 1) will be done across the 80 city members of the French WHO Healthy Cities Network. A database will be developed. This database will be used to select 6 city cases (first step in Work package B). Furthermore, drawing on the data from the 80 cities, city profiles will be identified and will contribute to the transferability of results (Work package D).

- Work package B (QUAL-quant): Characterization of the HiAP approach and governance and green space policies (*N* = 6)

**Table 1:** Goals, dimensions, methods and tools for work package A

Goal: • constitute a baseline database of each healthy city according to its socio-demographic, geographic, green spaces characteristics and characterize the HiAP approaches in the 80 cities • build city profiles according to its socio-demographic, geographic, green spaces characteristics and characterize the HiAP approaches in the 80 cities	
Dimensions analyzed	Methods and tools
Elements of description of each city that will be used as context factors concerning the demographic situation and dynamics, socio-economic heterogeneity, urban segregation (a dissimilarity index will be constructed by an aggregation of different indicator: unemployment rate, worker’s rate, higher education rate and median income at infra-communal scales)	Web-based data collection using the national socio-demographic base (INSEE. Data will be analyzed with Excel software
Information about urban green spaces (quantity, surface area, etc.) to appreciate the coverage of urban green spaces in each city (relevance of urban green space in the city, share of urban green space per inhabitant, etc.)	Web-based data collection using the national geographic and topographic base (IGN) and the participative Open Street Map data set will be used. Data will be analyzed with Excel software and mapped with Philcarto and ArcGis programs
HiAP approaches will be studied according to the degree of maturity drawing on the works of Storm et al. [51] and to the extent to which they address health inequities and green spaces in policy making	Two different online surveys: one for the elected officials and the practitioners responsible for the cities’ heath issues, and the other for the officials in charge of green space management

In order to develop the case studies, the aim is to select 6 city profiles based on socio-economic and urban green spaces characterization and a typology regarding the HIAP process in the cities and the inclusion of health into green spaces policy. This approach is based on the assumption that in cities with equivalent socio-economic characteristics, the maturity level of the HiAP process determines equity and health in green space management policy.

**Fig. 3:**
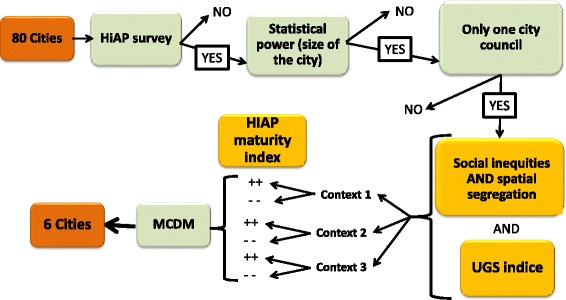
Selection levels to identify cities includes in the in depth study. *UGS: Urban green space. *MCDM: multiple criteria decision making

Two levels will used to select cities included in the in-depth study according to the city’s administrative organization, the response to the HiAP survey, the socio-economic heterogeneity and degree of spatial fragmentation, asynthetic indicator of urban green space availability and the *maturity of the HiAP process*. The selection levels are described in Fig. 3.

The sample of cities will present the 6 different profiles. Should these two levels of selection happen to be insufficient for selecting 6 city cases, we will apply a multiple-criteria decision-making approach using preference criteria based on the ELECTRE and PROMETHEE methods [44]. The Advisory Board will determine the number and nature of the criteria to put into the preference model.

Once the 6 cities are identified, the qualitative in-depth study for each city will be developed as presented in Table 2.

- Work package C (QUAL - QUANT) – Geographical characterization of green spaces and green spaces use (*N* = 6)

**Table 2:** Goals, dimensions, methods and tools for work package B

Goals: • Characterize the cities according to the HiAP process of each case: levels of governance implemented (internal, external) and the strategies related to health, policy making processes related to green spaces and urban planning (44,45) *(WP B1).* • Identify policy action relating to green spaces and intentions in terms of health and reducing health inequities *(WP B2)*		
	Dimensions analyzed	Methods and tools
*WP B1*	Combining different frameworks of HiAP and SIH assessment: - identification of the governance tools and the authorities involved - characterization of HiAP strategies [33]: cooperation, damage limitation, win-win situations and health-centered decision making - characterization of intersectoral governance in terms of coordination and sustainability [53] - characterization of the strategies aimed at reducing health inequities by using the Gradient Equity Lens [54] which describes, for every stage policy making, the strategies impacting on health inequities. Here, strategies such as “proportionate universalism”, targeting vulnerable populations [55–57] or intersectoral strategies will be particularly characterized.	• *document collection and review* Documents will be collected from municipal websites and during on-site visits. A content analysis based on the dimensions of Gradient Equity Lens tool [58] will be conducted. The modes of governance identified in the documents will be analyzed according to the theoretical framework of Baum and al. [52] and according to coordination and durability dimensions [53]. • *semi-structured interviews* In each selected city, we will identify municipal staff along with staff from other organizations including from the private sector who deal with health issues. This will be done through the French Healthy Cities Network using snowball sampling. Interviews will explore partnerships and collaboration on health issues, health inequities and green spaces policy and management i.e. accessibility, esthetics, amenities and/or maintenance. All the interviews will be recorded and transcribed. A content analysis [25] will be performed with the software N′ Vivo10 ©.
*WP B2*	We will analyze two major dimensions: policy and implementation relating to establishing new green spaces, spatial planning, redevelopment and use of green spaces with regard to health and reducing health inequities. We will focus on accessibility (distance, number, continuity, safety), esthetics (landscaping, perception) and features (infrastructure...) and will draw on the theoretical model by Roué Le Gall [59]. We will furthermore analyze the mechanisms behind policy action on health inequities. This will enable us to clearly demonstrate the logical model of the policy action.	• *a documentation review*: urban plans (municipal town planning documents) and green spaces intervention plans will be already collected in work package C. • *semi-structured interviews, on-site visits and participatory meetings*. The logical model will be built and validated during these meetings.

In order to characterize the green spaces and their use in each one of the cities, the in-depth study of the cities will be completed with this workpackage. The aims, dimensions and methods are presented in Tables 3 and 4.

- Work package D**:** Case study synthesis and transferability

**Table 3:** Goals, dimensions, methods and tools for work package C1

Goal: • Study spatial and geographical characteristics of the urban green spaces. This analysis will enable us to answer the following questions: what, where, how are urban green spaces distributed, managed and organized? Are there marked differences across the 6 cities in the type, location, distribution and nature of their green spaces? How can cities be characterized by their green spaces? *(WP C1).*		
	Dimensions analyzed	Methods and tools
*WP C1*	analyze the spatial distribution of green spaces in the city, the amenities, and the appropriateness with the social and demographic characteristics of cities and districts, their accessibility, configurations, nature and surface: - *Size, topography* (steeps slopes for example) and *type* (child recreation parks...) - *availability* (walkable travel times, signposts, number of entrances, number of bus stops existing within 200 m of a green space, etc.) - *Location* (spatial concentration vs. equitable spatial distribution of GS in towns for example) and *neighborhood types* (e.g. are they located in poor or rich districts, young or old populations?) - *Broader context* (are cities and their GS located near large national parks, the sea, mountains … or in a very dense and mineral area?)	Urban green spaces will be chosen in each of the six cities in order to ensure data representativity and comparability. These spaces will be identified by green space managers and municipal health departments as representing some form of strategy to reduce health inequities. Once these spaces have been identified, quantitative and qualitative data will be used for mapping and analysis: - IGN (BD-Topo base) and Open Street Map data to appraise the coverage of urban green space in each city (relevance of urban green space in the city, share of urban green space per inhabitant …). We will also measure the potential availability of urban green spaces potential availability using road network data and urban public network data - IGN (BD-Topo base) and Open Street Map data to appraise the location of each city and its green spaces in relation to important, or significant, natural places such as mountains, sea, and preserved national parks - Qualitative study results from observation in situ. Field data will be necessary for analyzing the urban green space type and topography, accessibility (number of entrances), etc. Finally, collected geographical data will be integrated into and processed by a geographical information system (SIG). The data will be used according to traditional spatial analysis methods at different scales (city, districts, and neighborhoods).

**Table 4:** Goals, dimensions, methods and tools for work package C2

Goal: • Observe physical features of green spaces and describe the use and non-use of urban green spaces identified by green space managers and municipal health departments as representing some form of strategy to reduce health inequities *(WP C2)*		
	Dimensions analyzed	Methods and tools
*WP C2*	For each city: seasonal use green spaces, (the main criteria for attractiveness), leisure use, surroundings, amenities, etc. This work package will complete the geographical data and help to characterize the use, esthetics and management of each urban green space selected. This will serve to enable different cities to share practices and enhance their innovation. For researchers, this will be an observational phase with knowledge transfer in mind	A qualitative study will combine *observation* in situ and *interviews* with green space users or non-users, and stakeholders involved in urban green space management and maintenance. - *Observations:* An observer will make observations in each selected city, at 4 different times per year in the different green spaces identified by the mapping (objective 2). We will explore items in order to characterize green space environments including: esthetics, equipment, leisure amenities, location, neighborhood, and users. - *User and non-user interviews*: Through on-site meetings with users and non-users living next to green spaces, interviews with residents could be planned in order to learn how they view the green space. Together, the interviews and observation periods at different times of the year will enable us to identify routine and occasional uses of public green spaces. - *Stakeholder interviews:* For each city, we will interview some key stakeholders of green space interventions (politics, NGOs, spatial planners, citizen committees, local organizations …) in order to better understand local green space policy, use and management. Finally, this study will also include *on-site visits with practitioners from the selected cities*. For each city observations will be completed by on-site visits with different stakeholders and policy makers concerned by the study.

The collection of cases will be cross-analyzed (WP D1). Each case study will deliver:

1. *a typology of cities regarding their local political system and HiAP governance* concerning green spaces and health inequities (results of the socio-political analysis) which will be crossed with the distribution of green spaces across the city (results of the spatial analysis). This typology will help to identify different city profiles
2. *a typology of green spaces for each city regarding their locations, representations, uses, amenities* (i.e. defined criteria during the study) which will be crossed with the level of use of green spaces.

In order to answer our main research question, those two types of typologies will be crossed to identify:

- for similar “green space” types and urban context but different “modes of governance”, which mode of governance seems to favor wellbeing and tackle the health inequities
- for similar “modes of governance” but different “green space” types and urban context, which configuration of green spaces seems to favor wellbeing and tackle health inequities.

The aim is to set out contextualized recommendations for all the network cities, based on the lessons from the 6 cases (WP D2).

We will first establish the profiles of the 80 cities according to 4 dimensions:

- Contextual factors: socio-demographic situation and dynamics, position of the city in the French Healthy Cities Network, location of the city in relation to important or significant natural places such as mountains, sea, and preserved national parks,
- socio-economic characteristics: the socio-economic heterogeneity and urban segregation,
- the HiAP approach’s degree of maturity,
- green space characteristics: size, nature, context and accessibility.

Secondly, we will compare the profiles identified with the components of HiAP governance suited to implementing green spaces in urban areas. For each profile we will identify the strengths and weaknesses with regard to HiAP governance for implementing green spaces suited to healthy living and for setting out contextualized guidelines for each case.

A methodology will be developed from this and disseminated widely. It will be sent to cities (including those outside the WHO network) to enable them to analyze their own possibilities for HiAP governance applied to green spaces in urban areas. The project includes various knowledge transfer modalities such as: the production of policy-briefs, local seminars and a national conference with local politicians and other policy makers. A collective publication similar to other produced by the WHO Healthy Cities Network [45] containing the most promising interventions will be part of the sharing of key messages at both local and national level. Tables 5 and 6 present the knowledge transfer strategic plan.

**Table 5:** Knowledge transfer strategic plan (part 1)

Type of production	To whom? (targeted audience)	Why? (Specific objectives)	How? (Strategies of KT)	With whom? (stakeholders)	What context?	Indicators of achievement
Diffusion to scientific peers						
1 – Oral communication and/or poster	Peers, community of research	Inform: present the results of the research and improve the approach Disseminate: the results - on the protocol - on the results Communicate: data on evidence-based intervention concerning the reduction of health inequities at a local level	Less interactive strategy: oral presentation; poster or power point Interactive strategy During oral communication – exchange with the scientific community	Team of research Team of the French network of healthy cities	National congress: SFSP International congress: EUPHA, IUHPE Seminar: Health and environment Academic value and scientific credibility Scientific value of the project	Number of communication oral or not/in France and at international
2 – Scientific papers	Peers, community of research	Disseminate: the results - on the protocol - on the results	Less interactive strategy Publication on peer-reviewed papers	Team of research		Impact factor of publication Number of published articles
3 – Research seminars	Peers, community of research	Exchange on methodology and results Improve research protocols	Interactive and structured strategy Exchanges with the scientific community and written production Less interactive strategy Dissemination of the minutes	Team of research	Scientific enhancement Close-up seminar Workshop– EUPHA, IUHPE	Seminar y/n Number of publications
4- Final research report	Peers, community of research Funders	Endorse accountability Justify the funds Disseminate results and knowledge	Less interactive strategy Disseminations to and beyond the institutions of the stakeholders of the project	Team of research	Scientific value of the project	Fund use and justification Accountability Number of publications

**Table 6:** Knowledge transfer strategic plan (part 2)

Type of production	To whom? (targeted audience)	Why? (Specific objectives)	How? (Strategies of KT)	With whom? (Stakeholders)	What context?	Indicators of achievement
Diffusion to practitioners						
5- Case study presentation	Selected cities, elected officials	Inform: present the results of the research and improve the approach Disseminate: the results - on the protocol - on the results Highlight the participation of cities	Short-term interactive strategy: exchanges decision-makers and elected officials Less interactive strategy Web-diffusion of the minutes	Network team Research team	Feedback information on results and interaction	Number of presentation
6 –Site visits	Selected cities	Exchange on practices between the ejected-official and stakeholders Disseminate innovation on green spaces, urban planning and governance Reinforce team-building	Less interactive strategy Individual case study giving to the selected cities Public dissemination of the final report	Network team: leader Research team (assist)	Feedback information on results and interaction Part of the research protocol	Number pf visits/number of participants
7- Publication of a practical guide by the French network of healthy cities and the EHESP	French Network of Healthy Cities Other French municipalities European network of Health cities	Exchange on practices between the ejected-official and stakeholders Disseminate innovation on green spaces, urban planning and governance	Less interactive strategy Dissemination of the guide and free access on the French Network of healthy cities web-site	Members of the board of the French Network of Healthy Cities Network team Research team	Document for dissemination to general public	Number of practical guide published and disseminated Number of downloads
8-Seminar French Network of Healthy Cities /Ehesp	Elected official, decision-makers Researchers on public health	Exchange on practices between the ejected-official and stakeholders Disseminate innovation on green spaces, urban planning and governance	Short-term interactive strategy exchange between participants during the seminar Continuous but less interactive strategy Creation of a short movie (5 min) about health and urban planning – available on line	Members of the board of the French Network of Healthy Cities Network team Research team	Communication through the French network of healthy cities and the other municipalities Improvement of knowledge about interventions on health inequities	Number of participants Number of papers Number of web-visits
9- Briefing notes HiAP/EV/Paysage	Elected official, decision-makers, practitioners Other French municipalities European network of Health cities	Disseminate innovation on green spaces, urban planning and governance	Continuous but less interactive strategy – briefing notes available for all, researchers and practitioners	Team project	Innovation on KT support	Number of downloading

## Discussion

This study protocol is designed to explore Health in All Policies (HiAP) as a way to help reduce health inequities through the use of urban green spaces. The research aims to produce a set of guidelines based on good practice at local and municipal level. However it is highly important for results to be replicable and for practitioners to adopt the new-found knowledge. This is challenging as results need to fit local contexts and be accepted by stakeholders. If public policy makers are to adopt these guidelines, they need to understand what they are for and how they can implement them within their own local contexts and practices. Transferring and applying results therefore means addressing contextual factors and understanding the constraints under which policy makers have to operate. One way to achieve this is to implement an approach that is both interventional and participative.

The GREENH-City project design comes under real life studies. There are recommended as a way of understanding the impact of population-level policies on health outcomes or health inequities [46, 47]. They differ from controlled experiments in that researchers do not manipulate any intervention data, but only observe them in real-life conditions. They involve a comprehensive approach to processes, which lends itself particularly well to observing policy processes [48, 49]. The research design is suited to studying municipal policy making and governance relating to health and green spaces, which is what we are looking to study. In this research therefore, the factors observed in-situ will be contextual factors specific to each case.

This approach enables us to identify, according to context, how a policy may have a bearing on health inequities. Contexts are an integral part of the analysis and they define the key elements for ensuring a replicable methodology. They enable us to understand the extent of health outcomes achieved by HiAP processes, and they act as policy levers to improve practices. By understanding what is replicable in another setting, we can adapt our guidelines and help ensure HiAP in urban areas can be applied to green spaces in new contexts.

GREENH-City is also about participative research in that it involves project beneficiaries (WHO Healthy Cities Network member cities) throughout. The cities involved are both areas of exploration and research partners. The participatory process is important to secure results appropriation and to improve their transferability. The French Healthy Cities Network will continue to engage with stakeholders in order to help ensure practical uptake of the results. Stakeholder involvement in the intervention design and implementation relating to green spaces is certainly a success factor for the interventions [50].

## Conclusion

This study covers a new area of research relating to urban policy on green spaces and its influence on social and territorial inequalities in health. The aim is to help improve people’s health and to act on those factors which influence chronic disease positively or negatively through living conditions. The study considers how the built environment may offer greater health outcomes and how green spaces are essential to wellbeing. This interventional research will be applied locally using a participative approach. It has a natural experiment design, addressing the specific contexts of each case study. A set of guidelines will be produced from this study so that other cities may enhance their green space policies and reduce health inequities.

## Additional file

Additional file 1: Annex 1 describing the objectives and methods by work package (WP). The notification of QUANT and QUAL depends on the nature of the method used. (DOCX 17 kb)

## Abbreviations

GREENH-City: GoveRnance for Equity, EnviroNment and Health in the City
HiAP: Health in All Policy
IGN: Institut National de l’Information géographique et forestière
INSEE: Institut National de la Statistique et des Etudes Economiques
MCDM: Multiple criteria decision making
QUAL: Qualitative
QUANT: Quantitative
UGS: Urban green space
WHO: World Health Organization
WP: Workpackage
