# Correction to: The GREENH-City interventional research protocol on health in all policies

**DOI:** 10.1186/s12889-017-4873-8

**Published:** 2017-11-06

**Authors:** Marion Porcherie, Zoé Vaillant, Emmanuelle Faure, Stéphane Rican, Jean Simos, Nicola Luca Cantoreggi, Zoé Heritage, Anne Roue Le Gall, Linda Cambon, Thierno Amadou Diallo, Eva Vidales, Jeanine Pommier

**Affiliations:** 1 EHESP –School of Public Health, Department of Social Sciences and Health, 15 avenue du Professeur Léon-Bernard - CS74312 -, 35043 Rennes cedex, France; 2 ARENES, (UMR/CNRS 6051), University of Rennes 1 Institut d’Etudes Politiques, 104 Boulevard de la Duchesse Anne, 35700 Rennes, France; 3 University of Paris-Nanterre, Ladyss - UMR 7533, 200 Avenue de la République, 92000 Nanterre, France; 4 Institute of Global Health, University of Geneva, Chemin des Mines 9, CH - 1202 Genève, Switzerland; 5 WHO French Healthy City Network, 15 avenue du Professeur Léon-Bernard - CS74312, 35043 Rennes, France; 6 EHESP –School of Public Health, Department of environmental and occupational health and sanitary engineering, 15 avenue du Professeur Léon-Bernard - CS74312, 35043 Rennes cedex, France; 7 EHESP –School of Public Health, INCA/EHESP Research Chaire in Cancer Prevention, Department of Social Sciences and Health, 15 avenue du Professeur Léon-Bernard - CS74312 -, 35043 Rennes cedex, France; 8 École supérieure d’aménagement du territoire et de développement régional– Université Laval, Pavillon Félix-Antoine-Savard, bureau FAS-1616, 2325, allée des Bibliothèques, Québec, QC G1V 0A6 Canada; 9 EHESP – National School of Public Health, Department of Social Sciences and Health, 15 avenue du Professeur Léon-Bernard - CS74312, 35043 Rennes cedex, France

## Correction

After publication of the article [1], it has been brought to our attention that in the original publication the third author’s name was spelt incorrectly. The correct spelling is “Emmanuelle Faure”. This was previously spelt as “Emmannuelle Faure”. The original article has been revised to reflect this.
