# Supplementary material for: The GREENH-City interventional research protocol on health in all policies
Source: BMC Public Health. 2017 Oct 18;17:820. doi: 10.1186/s12889-017-4812-8 (PMC5648502; doi:10.1186/s12889-017-4812-8)
Supplement: Additional file 1: — Annex 1 describing the objectives and methods by work package (WP). The notification of QUANT and QUAL depends on the nature of the method used. (DOCX 17 kb) [file 12889_2017_4812_MOESM1_ESM.docx]

| **Objectives** | **Method** | **Sample** | **Dimension analyzed** | **Tools** | **Deliverables** |
| --- | --- | --- | --- | --- | --- |
| **Baseline study** | ***WP A***  QUANT | 80 cities | Characterization of each city according to the socioeconomic context, surface area and distribution of green spaces | Secondary analysis of data collected on web bases | Database  City profiles |
|  |  |  | For each city: how HiAP is approached, how social inequalities are addressed in policy making and how health issues are integrated into green space policy | Questionnaires | City profiles |
| **1-** Understand how the cities implement the approach of health in all the policies, in order to take into account the social inequalities of health thanks to the political decisions related to green spaces | ***WP B***  QUAL-quant | 6 cities | Identification of 6 case studies | Analyses of the 80 cities database | Selection of 6 cities profiles |
|  |  |  | WP B1: Identification of the tools of governance  Identification of the HiAP strategies : cooperation, limitation of damages, win-win health in the center of the decisions ,  Intersectoral governance approach : coordination and durability  Strategies to act on the social determinants of health | Documentation collection: plans, programs, plans relative to the interventions of urban planning  Semi-structured interviews  On-site visit  Participatory meetings | Typology of cities according to the mode of governance and the interventions on the SIH  Description of the processes of the interventions produced by cities |
|  |  |  | WP B2: Green space policy across accessibility, aesthetic, management based on the Roué Le Gall et Potelon, framework (2014), Lachowycz et al (2013) and mechanisms on health and health inequities | Semi-structured interviews  On-site visit  Participatory meetings | Description of Green space policy |
| **2-** Describe and analyze the interventions produced and implemented within the cities on the green spaces from a geographical point of view, according to the socio-economic characteristics of the cities. | ***WP C***  QUAL  QUANT | 6 cities | WP C1: Geographical situation and place of green spaces by cities, neighborhood, , characterization of green spaces | Mapping (Cartography) / geographical analysis | Description of the characteristics and typology of green spaces by taking different criteria into account |
| **3**- Analyze the use and the contribution of green spaces on health and well-being of the inhabitants of the cities. |  |  | WP C2: Characterization of use and management of the green spaces, Lachowycz et al (2013), Hitchings, 2013 | Observational studies of green spaces (4 times/y)  Semi-structured interviews  On-site visit  Participatory meetings | Description of the physical characteristic of the green spaces as well as a typology of the uses and perception of health benefits different of different characteristics |
| ***Cross-case study*** | ***WP D1***  QUAL  QUANT | 6 cities | 1/- By a typology of the HiAP strategies 2/- By a characterization of the interventions on the green spaces 3/- By outcomes of the observation on the use of the green spaces. | Transversal analysis of the 6 cases studies | City profiles  Recommendations of HiAP implementation |
| ***Recommendations and transferability of results*** | ***WP D2***  QUAL | All the cities | Convergence factors between cities regarding  HiAP profile, greens spaces characteristics, socioeconomics profiles | Databases (deliverable Step 0)  Socioeconomic synthetic indicators | Applied recommendations to similar cities that have been identified at step 0 |
